# The productivity tax of new office concepts: a comparative review of open-plan offices, activity-based working, and single-office concepts

**DOI:** 10.1007/s11301-022-00316-2

**Published:** 2023-01-05

**Authors:** Andrea Gerlitz, Marcel Hülsbeck

**Affiliations:** Witten Institute of Family Business, WIFU-Foundation Chair of Family Business Management, Universität Witten/Herdecke, Alfred-Herrhausen-Str. 48, 58448 Witten, Germany

**Keywords:** Productivity tax, Employee, Satisfaction, Performance, Office concept, Open-plan office, Activity-based working, Single office

## Abstract

This systematic review critically analyzes the relationship between office concepts and individual and organizational performance. Based on the current literature, we identify seven key dimensions to guide our understanding: office concept, work, personality fit, satisfaction, health, control paradigm, and enabling paradigm. Our systematic search yielded 429 published papers on office concepts and performance between 2005 and 2022. Rigorous selection criteria narrowed them down to 46 empirical articles included in this analysis. The results show that activity-based working not only negatively impacts performance but also affects employee satisfaction and health. Open-plan offices can reduce real-estate costs but lead to lower performance levels, thereby imposing a tax on productivity which outweighs the initial cost savings. Activity-based working has the potential to enhance collaboration and interaction but is dependent on a professional and proactive management. In most cases, especially for knowledge workers, the single office turns out to be the environment in which employees show the best individual and organizational performance.

## Introduction

The “new world of work” causes fundamental changes (Aroles et al. 2019) that lead to reorganizations of work and workplaces (Okhuysen et al. 2013; Schmid and Dowling 2020). These have consequences for office layouts. Scholars distinguish several dimensions of office concepts that affect employees' behavior and subsequent individual and organizational performance (Węziak-Białowolska et al. 2018). When deciding on the implementation of new office concepts, corporate real estate and facility management mostly consider real estate costs (Appel-Meulenbroek et al. 2018; de Vries et al. 2008) as potential benefits are complex and therefore hard to assess (Feige et al. 2013). At the same time, boundaries in the office environment are being removed to foster interaction and collaboration (Bernstein and Turban 2018). Although the theory on how removing boundaries affects human behavior is divided (Ward et al. 2017). Although the social facilitation hypothesis, represented by sociologists, suggests that removing boundaries may foster collaboration and collective intelligence (Geen and Gange 1977), organizational scholars, mainly social and environmental psychologists (Taylor 1975), draw an opposite conclusion. Removing boundaries decreases the occupants' collaboration (Kim and De Dear 2013; Brennan et al. 2002; Bosch‐Sijtsema et al. 2010). Negative aspects that scholars discuss regarding unbounded office concepts include levels of distraction and a decrease in satisfaction and health, which result in a loss of productivity. These accumulated adverse effects cause stress and perceived pressure on employees, resulting in less knowledge sharing and collaboration (Škerlavaj et al. 2018). However, defining the optimal office concept for a given organization is not a trivial task. The prevalent focus on real estate costs may result in a productivity tax that outweighs the short-term cost-benefits. Still, open office concepts enjoy an excellent reputation. Companies like Google, with their mission to “create the happiest, most productive workplace in the world”, made these office concepts popular worldwide. However, empirical evidence points to the risk that employees feel dehumanized after relocation to a new office concept (Taskin et al. 2019). The following three office concepts dominate the office environment with specific characteristics. *Single and shared offices* are enclosed private rooms for one to three individuals (De Been and Beijer 2014) with additional enclosed meeting rooms, as well as shared facilities for printers, etc. In general, occupants of single and shared offices can control the heating and lighting per room. *Open-plan offices (OPOs)* are large unbounded open spaces where many employees have their workstations. Often transparent materials (e.g., glass) are used as design elements to create openness. Generally, OPO have a central climate and lighting system (De Been and Beijer 2014). *Activity-based working* (*ABW*), sometimes also called flex office, is also an unbounded large open office, but without fixed/assigned workstations. There are fewer individual workstations provided than employees working in the facility. Out of a variety of work settings (for example, areas for collaborative work), employees need to choose where to work according to their preferences and daily activities. After leaving a workstation, employees are expected to clean their desks (De Been and Beijer 2014). Taking into account the diversity of these office concepts, it has become necessary to develop a clear and holistic understanding of the relationship between office concepts and the individual and organizational performance aspects. To investigate these performance aspects, it is necessary to understand the differences between office concepts and why they might vary in their performance. Scholars treat performance as a multifaceted phenomenon (Carroll 1979; Chandler and Hanks 1993; Luo et al. 2012). Within the research stream on office concepts, performance is ill defined as a multi-dimensional concept that comprises quantitative and qualitative performance on the individual as well as on the organizational level (Kämpf-Dern and Konkol 2017; De Croon et al. 2005). Therefore, we use the term performance as an umbrella term that embraces the eclecticism of current research on office concepts.

This systematic integrative literature review (SLR) aims to structure the eclectic state of the literature by dimensions in this growing field and offer advice for practitioners. This interdisciplinary research covers psychology (Seddigh et al. 2015), real estate management (Haynes 2007), architecture (Megahed and Ghoneim 2020), human resource management (Taskin et al. 2019), engineering (Veitch et al. 2007) and health (Pejtersen et al. 2011; Zhang 2020). However, an integrative model is lacking. So far, many reviews have been conducted (Gjerland et al. 2019; Richardson et al. 2017; Engelen et al. 2019; De Croon et al. 2005). However, these reviews examined either one specific office setting, such as ABW (Engelen et al. 2019), or one particular performance, such as health (Richardson et al. 2017; De Croon et al. 2005), or conducted a scoping review that does not analyze in depth why and how office concepts impact performance but identified research gaps (Gjerland et al. 2019). Therefore, we follow the urgent call by Gjerland et al. (2019) to holistically examine the relationship between office concepts and various other categories such as level of distraction, privacy, satisfaction, and performance. Furthermore, we integrate employee satisfaction and health as a largely neglected category (De Croon et al. 2005; Richardson et al. 2017) in the ongoing discussion on the three most important office concepts: OPO, ABW, and single office. This led to the following research question: How do these three different office concepts affect individual and organizational performance?

This paper makes the following value-added contributions. First, it adds to the debate office concepts by providing a detailed and critical look at their impact on satisfaction and performance. Second, findings are visualized. Third, this article gives concrete suggestions for practitioners to view office space not merely as real estate costs but as an environment that may foster or hinder employees' satisfaction and productivity. Fourth, our research agenda will enhance and guide future research.

This paper is organized as follows. The next section describes the methodology used for this SLR. In the third section, we analyze the results of the literature based on dimensions of prior literature and conclude with our visualization of results. In the concluding section, we summarize the main results of this SLR, acknowledge limitations, and discuss promising avenues for future research. We conclude with practical implications.

## Method

This SLR aims to analyze the relationship between office concept (single office, OPO, or ABW) and individual and organizational performance. A systematic literature review (SLR) was conducted to create a transparent data collection and synthesis process that maximizes the level of objectivity and reproducibility (Tranfield et al. 2003) and minimizes possible authors' bias in the selection of literature to identify the status quo of existing literature as well as generate new insights (Pittaway et al. 2014) that may support decisions in research and practice (Briner and Denyer 2012). We followed the three stages of a SLR: planning the review, conducting the review, and reporting the findings (Kraus et al. 2020; Pittaway et al. 2014). We based the inclusion criteria for this SLR on several a priori considerations. To ensure the reliability of the empirical analyses in each article, only peer-reviewed articles are included (Bouncken et al. 2015) that are in English language and empirically evaluate office layouts (namely “open-plan office”, “activity-based working”, “single or shared offices”). Researchers and practitioners use a wide range of different office layout descriptions. However, after consulting with experts (Kraus et al. 2020) and analyzing previous reviews (Gjerland et al. 2019; Richardson et al. 2017; Engelen et al. 2019; De Croon et al. 2005), we identified these three office layouts to be the most relevant. These strict inclusion criteria seemed necessary to strive for an evidence-based model (Webster and Watson 2002).

Based on the insights of the seminal review by De Croon et al. (2005), we sample articles from 2005 to 2022. In 2005 the New Work Movement experienced a renewal. From this time onwards, office concepts such as the OPO and the ABW have played an increasing role and are designed and implemented worldwide. A new stream of research studies the effects and implications of office concepts. This SLR analyzes these more recent studies. The search was carried out in February 2022 on the leading scientific research database EBSCO (narrowed down to Business Source Premier, EconLit, Psychology and Behavioral Science Collection, APA Psychology Articles, and Medline) to identify relevant, high-quality studies and to ensure an exhaustive search across disciplines. A literature search was conducted threefold. First, we searched on EBSCO for articles whose titles, abstracts, or keywords contained “open-plan office” (short: OPO). It resulted in 170 matches. We then combined them with (AND) “productivity or efficiency or performance” (which resulted in 85 papers). Because the terms “productivity”, “efficiency,” and “performance” are not clearly defined and are often used interchangeably, we included all three terms in our search. In the second stage, we searched for articles whose titles, abstracts, or keywords contained “activity-based working” (102 results) and also combined them with (AND) “productivity or efficiency or performance” (35 results). In the last stage, we searched articles concerning “office concepts” or “work environments” (121 results) and combined them also with (AND) “productivity or efficiency or performance” (51 results). This initial search yielded 429 published articles. The database search was supplemented by additional articles identified through reference checks and expert interviews with two scholars. This resulted in 4 additional articles covering OPO, 10 additional articles on ABW, and 1 article added to our search of “office concepts” or “work environment”. We cleaned the raw data in a four-step procedure. First, we manually removed all duplicates. Second, all non-English articles were manually removed. Third, only studies that were conducted among adults who perform paid office work were included. Fourth, the authors read all paper titles and abstracts. We focused on papers that empirically investigate the relationship between office concepts and employee behavior and organizational performance. We excluded articles that (a) lack empirical primary data, (b) dealt with related fields but did not meet the research objective, (c) did not measure any outcomes, (d) focused on air quality, (e) focused on pathogenic effects of the office environment on pre-existing diseases (e.g., cancer, diabetes), and (f) focused only on gender differences. The remaining 92 articles were thoroughly read and verified. Overall, 46 papers meet the specific inclusion criteria for this SLR (see Table 1). The first author kept a review protocol to capture the evolution of the gained insights.

**Table 1:** Search strategy and paper inclusion

Positive selection criteria		
Language	English	
Publication	Academic Journals	
Date	Papers published between 2005 and 2022	
Data base	1. EBSCO	
	1.1 Business Source Premier	
	1.2 EconLit	
	1.3 Psychology and Behavior Science Collection	
	1.4 APA Psychology Articles	
	1.5 MEDLINE	
	2. Additional relevant articles	
1	Query:“Open Plan Office”	Result
	Titles	170
	AND “productivity or efficiency or performance”	85
	Relevant title and abstract	26
	Relevant after reading	18
	Additional relevant articles	4
	Total relevant articles after reading	22
II	Query:“Activity based working ”	Result
	Titles	102
	AND “productivity or efficiency or performance”	35
	Relevant title and abstract	24
	Relevant after reading	6
	Additional relevant articles	10
	Total relevant articles after reading	16
III	Query: “Office concepts” or “work environment”	Result
	Titles	121
	AND “productivity or efficiency or performance”	51
	Relevant title and abstract	14
	Relevant after reading	7
	Additional relevant articles	1
	Total relevant articles after reading	8
	Papers included in this review	46

*Analytical phase:* We analyzed previous reviews and merged the dimensions used resulting in the following seven: office concept, work, personality fit, satisfaction, health, control paradigm, and enabling paradigm. In our analysis, we differentiate between individual performance based on subjective effects (personality fit, satisfaction, health, perceived productivity) and organizational performance (real estate cost, labor productivity).

**Fig. 1:**
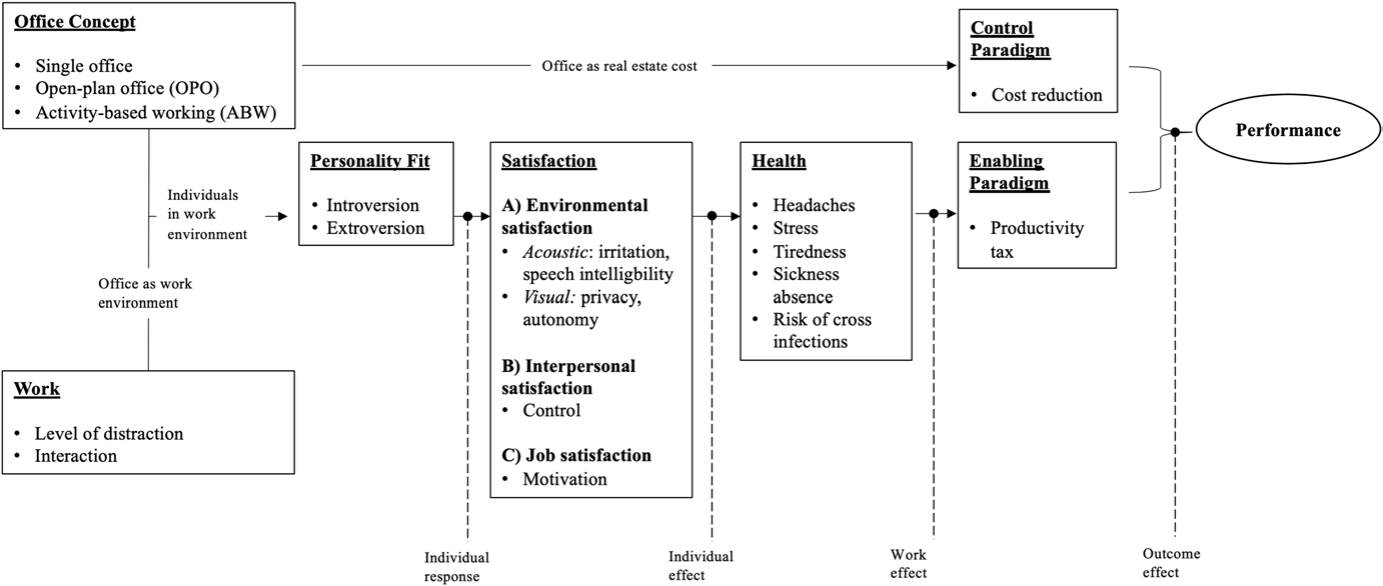
Visualization of results

*The office concept,* as one of our primary dimensions, refers to the workplace design and type of boundaries in an office (De Croon et al. 2005). The office concept influences how workspaces within a room are designed, where employees carry out their work. For this SLR, we include the following three different office concepts: single office (shared room office), OPO, and ABW. The dimension “work” comprises two variables: *level of distraction and interaction*. Depending on a given work task, workers are receptive to higher levels of distraction and require various levels of interaction. The level of distraction is the level of disturbance within a given work environment. The acceptable noise level varies depending on the cognitive workload (De Croon et al. 2005). Some tasks require quiet work zones to perform, while others, for example, repetitive tasks, can be fulfilled with higher background noise. *Interaction* defines the required amount of collaboration that results in background noise. *Personality fit* describes how different personality traits affect the perception of and satisfaction with office concepts (Ellwart and Schulze 2009). For example, an introverted employee may prefer a personal and quiet workspace, while an extrovert may enjoy coworking with others within a room (Wadu Mesthrige and Chiang Yat 2019). *Personality fit* influences employee satisfaction, health, and performance. Office concepts are linked to work and personality fit and affect the *satisfaction* with environmental (visual, acoustic, temperature), interpersonal (interaction with coworkers) and overall job aspects. While satisfaction focuses on short-term effects, *health* looks at the long-term effects. At best, occupants feel healthy and flourish; at worst, a dissatisfying work environment negatively impacts occupants' health (De Croon et al. 2005) and their motivation. *Enabling paradigm* takes a long-term perspective. It is a human-centered approach that shows the relationship between office concepts and performance (Haynes 2007) by incorporating dimensions such as work, personality fit, satisfaction, and health. The direct impact of a chosen office concept on real estate cost is called the *control paradigm*. It focuses on direct monetary efficiency but neglects to mediate variables that may lead to a productivity tax (Haynes 2007). Based on these categories, we analyzed and structured the data for our SLR. Within the final phase of conducting a review, the synthesis of findings is represented (Sect. 3). We followed the advice of Fisch and Block (2018) to conclude the findings section with a conceptual model (see Fig. 1).

## Analysis

In this section, we provide an analysis of the 46 reviewed papers and show the influence of office concepts on the other dimensions. A summary description of all reviewed articles is provided in the Appendix of this paper. First, we briefly introduce the different office concepts. Second, we analyze the dimension “work” by looking closer at the level of distractions and interaction. Third, we consider the effects of personality fit. Fourth, we evaluate employee satisfaction with the environment, interaction, and job. Fifth, we examine the influence on health. Sixth, we examine the effect of enabling paradigm on performance, and finally, seventh, we look at the cost paradigm. Each aspect is analyzed with respect to the specific office concept. We conclude each section with a summary.

### Office concepts

The literature does not use a single common terminology for office types. To align the language of different researchers, we developed the following taxonomy to define the different office concepts based on Duffy and Powell (1997) and Danielsson and Bodin (2008).

### Work: Sometimes it is distraction, sometimes it is interaction!

#### Level of Distraction

A recurring theme in the reviewed studies is noise-induced distraction. When speech intelligibility cannot be ensured, occupants feel distracted, and their satisfaction and performance diminish. Appel-Meulenbroek et al. (2021) studied occupants' coping strategies for different noise sources. Their findings show that choosing a specific coping strategy depends on the origin of the noise; for instance, when the noise source is near one's desk, occupants use the avoidance strategy by interrupting their work or trying to be quieter. However, the stress level will remain when trying to ignore the noise. Göçer et al. (2019) compared higher and lower performing OPOs and found noise as one predictor of lower performance. As distractions lead to greater stress and frustration, more errors are made (Mark et al. 2008), and collaboration is reduced (Škerlavaj et al. 2018). Noise appears as a consistently reported problem in OPO; see Hedge (1982). Wadu Mesthrige and Chiang Yat (2019) show that interruptions, overcrowding, and noise negatively influence the occupants' performance in OPO. Roskams and Haynes (2020) show that reducing the level of distraction results in higher productivity, which is in line with Roelofsen (2008). They also find that lower perceived distraction is associated with higher psychological comfort and enthusiasm, which is consistent with previous studies (Haynes 2008; Veitch et al. 2007; Candido et al. 2016).

Roelofsen (2008) states that in an office where potentially disturbing conversations take place the ideal noise level should be around 45 dB (A) and never be higher than 48 dB (A). Furthermore, noise can lead to working pressure, cause fatigue, depression, and reduces overall satisfaction (Jahncke et al. 2011; Sander et al. 2021). Personality and work tasks influence how employees perceive the disturbance level (Oseland and Hodsman 2020; Roelofsen 2008). Banbury and Berry (2005) found that 99% of their participants report concentration problems due to background noise from other employees. They also show that noise distraction is higher in OPO than in single offices. Pejtersen et al. (2006); De Been and Beijer (2014); Kaarlela-Tuomaala et al. (2009), and Kim and De Dear (2013) provide further evidence that the distraction level in single offices is the lowest. To cope with noise distractions in OPO, quiet work areas are a needed remedy (Chacon Vega et al. 2020; Chadburn et al. 2017; Roelofsen 2008; Seddigh et al. 2015). Employees moving from OPO to ABW perceive better conditions for concentration (Blok et al. 2009; Candido et al. 2019; Haynes et al. 2019; Rolfö et al. 2018). But distraction still play a role in ABW (Rolfö et al. 2018). It leads to less concentration (Keeling et al. 2015) and therefore less productivity (Wadu Mesthrige and Chiang Yat 2019) compared to single offices. Roelofsen (2008) recommends incorporating smaller closed office spaces to better cope with noise distraction. Noise distraction is a widespread problem in open office environments, especially in OPO, but also in ABW. The distraction level depends on the job role and personality and affects the occupants' performance. A growing number of employees are looking for quiet work areas. In a world where knowledge workers are in high demand, companies may be well recommended to reconsider OPO as the dominant office concept.

#### Interaction

Interaction is linked to noise and distraction because it can be seen as its counterpart. New office concepts like OPO and ABW have been designed to facilitate interaction, but face-to-face communication decreases in OPO environments (Brennan et al. 2002; Sailer and Thomas 2021). Bernstein and Turban (2018) examine how human interaction patterns change because of the architectural shift from a traditional office to an OPO. Gathering data by tracking face-to-face interaction with wearable sociometric devices and gaining data from electronic communication servers, including e-mail and instant messaging, they conducted two intervention-based field studies at corporate headquarters. Their results show that workers in unbounded offices reduce face-to-face interaction by 70%. The researchers observe that the employees try to isolate themselves as best as possible, pretending to be remarkably busy. In addition, the occupants feel observed and transparent. As a result, they choose electronic communication tools. When dealing with complex cognitive tasks that require deep work, irrelevant environmental stressors such as undesired interaction should be avoided to enable the occupants' performance. Chadburn et al. (2017) show that knowledge workers opt for a flexible range of office environments that offer room for quiet concentration work as well as areas for collaborative tasks. The ABW concept allows one to balance these needs (Keeling et al. 2015).

When experiencing a shift from OPO to ABW, employees notice an increase in interaction (Divett 2020; Blok et al. 2009; Haynes et al. 2019). Those with the autonomy to decide where and when to work perform better (Fincke et al. 2020). According to Rolfö et al. (2018), a proper time and space management system is indispensable for any ABW. Rolfö et al. (2018) find that a lack of communication and rules will lead to high people-to-workstation ratios. Problematic situations occur when a workspace is unsuitable for a given activity and team members do not find workstations close to each other. As a result, teams are scattered all over the place due to overcrowding and cannot collaborate as intended. Furthermore, participants report insufficient amounts of arrangements to have a collaborative gathering and that this lowers their performance. The architectural solution ABW does not automatically result in human interaction. The ABW setting requires rules, time, and space management to foster collaboration (Rolfö et al. 2018). Interestingly, two studies conducted in Teheran and Malaysia came to different results. Samani et al. (2017) studied employees in creative mobile industries (programmers and designers) in Malaysia. The findings show an increase in social interaction and creative output in OPO. A similar study from Teheran concludes that OPO increases interaction and employees experience a more democratic working culture (Samani and Alavi 2020b). The contradictory results of these two studies appear to be related to cultural differences. Although models that describe cultural differences have their limits in revealing in depth the cultural complexity of a country (Sure 2017), they can offer the first understanding. The Hofstede cultural distance model shows that power distance plays a significant role in Malaysia (100), is essential in Iran (58), and is less important in the UK and Germany (35) (Hofstede 2021). That leads to the assumption that the experience of open office concepts is modulated by culture. Bouncken et al. (2016) show that multicultural teams have the potential to increase innovation and creativity but likely struggle with different working and communication styles.

The physical office environment can trigger processes that foster personal interactions among employees and the quality of these relationships in the work context with respect to social and task-oriented bonds (Khazanchi et al. 2018), which may positively influence performance. Depending on the office concept, these interactions may become unintended background noise that distracts other co-workers and reduce their performance. Managers can influence with the implementation of office concepts the quality of the social interaction among their employees. All actors should be involved and participate in this process (Vereycken et al. 2021). ABW requires more conscious and deliberate planning than other office concepts. Sailer and Thomas (2021) conclude that the most unplanned interaction takes place in single/shared offices because workers in single or shared offices visit each other spontaneously. These spontaneous interactions occur more often because, unlike in OPO, other occupants are not affected by these interactions. Interaction.

### Personality fit: different strokes for different folks!

Individual differences, such as personality traits, influence how individuals perceive their work environment and how they perform. Wadu Mesthrige and Chiang Yat (2019) find that OPOs only suit extroverts but are not a good fit for introverts and highly sensitive people. In general, introverts prefer to have their own dedicated workspace. In contrast, extroverts are generally open to OPO concepts. This study also shows that conservative workers reject working in an OPO. These results are in line with the findings of Oseland and Hodsman (2020), who show that the performance of introverts is lower in an OPO setting. Going deeper into the behavior of different personalities in an open office setting, Appel-Meulenbroek et al. (2021) find that high extroverts tend to be quieter, medium extroverts are more likely to interrupt their work, while high neurotic choose to continue their work at home (if possible). Each office concept requires a specific way to deal with its unique atmosphere, which resonates with the personalities of the occupants, so that a given office concept may improve or reduce performance.

### Satisfaction: a place to call my own!

Scholars have been studying the psychological outcomes of office concepts, especially satisfaction. The reviewed studies focus on three aspects of satisfaction: environmental, interpersonal, and job. Roskams and Haynes (2020) find that reducing the level of disturbance improves satisfaction in OPO, which leads to greater environmental comfort and increased productivity. Another factor that increases satisfaction within an OPO setting is personal control over one's workspace (Ahmadpoor Samani et al. 2017). Two studies show that satisfaction with the work environment decreases after relocating from single offices to an OPO (Kim and De Dear 2013; Bergström et al. 2015), which is in line with Oldham and Brass (1979), who show that motivation decreases among employees who relocate from a single office or shared office to an OPO. They point out that motivation is related to interaction with colleagues and overall job satisfaction. Danielsson and Bodin (2008) concluded that job satisfaction is lowest in the OPO setting, which supports the evidence of other studies reviewed above, so we must conclude that OPO has a negative impact on satisfaction. A well-managed ABW gives employees autonomy to choose a workspace and therefore gives them control over privacy and noise, increasing employee satisfaction. For example, employees report an increase in satisfaction when moving into ABW with respect to auditory privacy and background noise (Rolfö et al. 2018). Other scholars have reported equivalent results (Candido et al. 2019; Divett 2020). Furthermore, occupants of ABW report that they like the new aesthetics but criticize the time and space management (Rolfö et al. 2018); see section “interaction” of this paper. As ABW does not allow for personal workstations, time and space management are crucial to ensure that work can be done. Employees complained that it is very hard to find an appropriate office space unless they come very early in the morning (Engelen et al. 2019). Additionally, Taskin et al. (2019) show that new employees are initially enthusiastic about the typical ABW environment but find themselves disoriented after working there for some time because they cannot easily locate relevant co-workers to address their questions. These two studies show that ABW occupants find the design stylish or appealing at first glance, but they also reveal the major problems of working in a flexible environment. In traditional settings, each person has a designated workstation which is often located according to hierarchy. An ABW is solely based on current tasks, hence not allowing a straightforward way to maneuver across the office floor. ABW is associated with a loss of orientation that leads to an overall experience of dissatisfaction. There is a trend these days to put a lot of ambiance and funny props in ABW. But Appel-Meulenbroek et al. (2011) show in their study that ABW should not incorporate too much of it because these elements are often unpractical and occupants refuse to use them. Instead, office environments should offer their occupants opportunities to personalize their workstations (Arundell et al. 2018; De Been and Beijer 2014; Candido et al. 2021; Kaarlela-Tuomaala et al. 2009; Gorgievski et al. 2010; Sidorenkov et al. 2022). Taskin et al. (2019) show that anonymous office designs of OPO and ABW make employees feel dehumanized, dispossessed, abandoned, and injuncted to adapt the modern behavior, which causes ill-being and dissatisfaction with the office environment. Therefore, employee satisfaction is influenced by the physical characteristics of the work environment and the implementation process (Brunia et al. 2016; Candido et al. 2021). Candido et al. (2021) show that interior design and physical configuration play a crucial role in employee satisfaction and productivity. They conclude that if ABW is well implemented and well-designed, it will fit many organizations across different industries. Satisfaction is rated lowest in OPO. In ABW, satisfaction is limited by its implementation and management; however, occupants have personal control in ABW, which adds to its overall appreciation. However, employee satisfaction is still highest in single or shared offices and lowest in OPO. It appears that employees who feel at home at work are more satisfied and hence perform better.

### Health: flourish or perish!

The reviewed studies of office concepts show mixed results on health. Moving from an individual office to OPO has negative effects on health. Bergström et al. (2015) show that after 12 months of working in the new OPO environment, employees' feeling of health deteriorated. This study investigates the long-term health effects of employees after three, six, and twelve months. This study measures “intrapersonal characteristics” (for example, feeling stressed) and “interactive function” (for example, feeling supported by colleagues) using the Salutogenic Health Indicator Scale, a test instrument that covers physical, mental, and social well-being. The rated health perception deteriorated. After three months, the differences are significantly worse; at six months, no significance can be measured, but at twelve months, perceived health deteriorates significantly. Bergström et al. (2015) see a possible explanation for the time when the enquiry after six months took place: it was just after the prime vacation time in summer. This may also have influenced the workers’ perceived health of workers. Another reason is that the employees adapted after a half-year of working in the new office environment. An acclimatization phase that followed an initial culture shock can be recognized after 3 months. Then, after 6 months, this effect stabilizes. If this effect were just a culture shock, recovery was to be expected after 6 or 12 months. But we see from their results that perceived health is even worse after 1year. Therefore, one can isolate the effect and one can observe a longitudinal negative effect on health in an OPO. Not being able to control environmental conditions such as air quality, temperature, humidity, noise, and lighting has a negative effect on health (Mulville et al. 2016). Mulville et al. (2016) show that behavior influences health; see also (Haynes 2007). Employees who take breaks more often suffer less from headaches and noise distractions. People take breaks when they feel exhausted. Feeling exhausted may stem from two leading causes: negative effects of the work environment, e.g., bad air, high noise, etc., or the desire to take back personal control. Danielsson and Bodin (2008) show that personal control is a significant indicator of preventing mental stress and strengthening health. In both cases, occupants try to counter the negative effect of the work environment and do something to improve their health.

As the OPO concept has adverse effects on workers' health, the new concept of ABW addresses these issues by giving employees control over where and when they prefer to work and by stimulating physical activity (Engelen et al. 2019), which nudges employees towards a healthier lifestyle. Candido et al. (2019) find that perceived health improves after relocation from an OPO to an ABW environment. Their post-relocation results show that occupants significantly reduce their sedentary time. Foley et al. (2016) conducted a similar study but found that breaks or step counts do not change significantly after relocation from OPO to ABW. Another pre-post study by Arundell et al. (2018) focusing on the shift from a traditional office to ABW reveals no significant improvements in sedentary time. Occupants describe an increase in incidental movements for job-related communication. Furthermore, this study also examines the eating behaviors (which no other study has done before). They concluded that ABW leads to healthier eating behavior. However, food vending machines were not available in the new office environment and a new policy banned eating at desks. The workers ate together in designated areas which increased peer pressure to eat healthier. It is not only the design of the office space that fosters a healthier lifestyle, but a new policy that is enforced and implemented at the same time with the new office concept. Appel-Meulenbroek et al. (2011) concluded in their study, which they conducted in four service organizations in The Netherlands, that implementation is key to success. ABW is more than a floor plan but requires a policy of use to ensure that workers benefit from it. If not properly implemented, ABW results in dissatisfaction and increased illness. Danielsson and Bodin (2008) examined seven distinct types of offices’ influences on health and satisfaction. Validated questionnaires were used, including self-reported sick leave, general health, and physiological and psychological problems of the occupants. The lowest health status was found in small and small OPO, and the best health status was found in single offices and ABW. Pejtersen et al. (2011) came to comparable results. They find that OPO occupants have a significantly higher number of sick leaves than those in single offices and suggest five mechanisms to explain the increased sickness absence rates in OPO: These might be (1) noise, (2) air quality, (3) spread of infections, (4) lack of privacy, and (5) lower autonomy.

Based on insights from the COVID-19 pandemic, the OPO concept may need to be reconsidered as high office density increases the risk of cross infections (Zhang 2020; Samani and Alavi 2020a) and could lead to a reversal of the current trending OPO (Megahed and Ghoneim 2020). To conclude, long-term health effects have been studied by a few scholars. The impact of office concepts on health, and therefore indirectly performance, appears to be strong. The data of this SLR suggest that OPO is the least healthy workspace; ABW motivates employees to do physical activities, which has a positive health effect that is also reflected in their performance. But the healthiest office work environment appears to be the single office.

### Enabling paradigm: make a workplace work!

Depending on the industry, labor costs make up about 80% of the total cost, and real estate costs account on average for only 10%, shows a study of multinational companies (Pfnür 2013). Thus, savings in real estate costs are relatively small in comparison to costs that are caused by a decrease in productivity through these office concepts (Appel-Meulenbroek et al. 2018; Haynes 2008): “Just a 2% decline in productivity can wipe out a tenant's cost savings (…)” (Newmark Grubb and Frank 2016). This leverage effect is dubbed the “productivity tax”. As an example: There are $550B annual losses in the US productivity of employees (Gallup 2013), as productivity is a matter of enabling workers to do their tasks. The critical drivers of productivity are team interaction and decision making (Divett 2020). Employees interact more and make better decisions when their overall satisfaction is ensured. Ahmadpoor Samani et al. (2017) and Haapakangas et al. (2018) show in their studies that supporting employees' satisfaction and improving environmental comfort motivate them to perform better. Göçer et al. (2019) find that individual space and personal control are the strongest predictors of productivity. These findings are in line with Herzberg's theory of motivation. In summary, these results show that the performance depends on the work environment and work conditions. To increase productivity, companies should implement appropriate workspaces that enable employees to master their specific work in an atmosphere that enhances individual satisfaction. As all other measures are lowest at OPO, so is productivity, as research by Bernstein and Turban (2018); Bergström et al. (2015); Ahmadpoor Samani et al. (2017); Roelofsen (2008); Roskams and Haynes (2020) shows. A shift from OPO to ABW has a positive impact mirrored by employee performance. Candido et al. (2019); Blok et al. (2009); Divett (2020) show that productivity increases when occupants move from OPO to ABW, but Rolfö et al. (2018) conclude that there is no change in productivity. Arundell et al. (2018) investigate the transformation from traditional offices to ABW and find a decrease in productivity. The single office outperforms OPO and ABW in all aspects, as studies from Pauls and Lütke Lanfer (2017); Krupper (2011); Danielsson and Bodin (2008); De Been and Beijer (2014); Taskin et al. (2019); Sailer and Thomas (2021) reveal.

### Control paradigm: what counts may not be what accounting counts!

Rising real estate costs led to a reassessment of office space occupancy (Węziak-Białowolska et al. 2018). Cost reduction is the most obvious rationale for transforming into OPO (Haynes 2007; Brunia et al. 2016; Baldry and Barnes 2012). As Blok et al. (2009); Parker (2016) show, ABW is even more cost-efficient than an OPO. Cutting real estate costs has a direct impact on performance. However, in the long run, the drawbacks of this short-term perspective become obvious.

Implementing office concepts plays an essential role in overall corporate success. Optimized office concepts can increase employee productivity by up to 13% (Pfnür 2013). Furthermore, companies can set a signal by incorporating attractive workplaces in the “war of talents” (Pfnür 2013; Jurecic 2020), which plays an essential role in employee retention and recruitment. The results of the study by Bauer (2014) show that being able to work self-determined positively impacts the work-life balance, resulting in higher motivation and higher overall performance. This may ultimately lead to a new human-centered approach to management in which employees are empowered to participate early in the process (Edwards and Ramirez 2016; Vereycken et al. 2021).

## Discussion

### Conclusion

This SLR studies the relationship between office concept (single office, OPO, or ABW) and individual and organizational aspects (e.g., satisfaction, health, and performance). Single office scores best concerning levels of distraction, interaction, satisfaction, health, and performance levels and is followed by ABW, which has a higher level of disturbance. The OPO scores the lowest. The rationale for implementing OPO and ABW is cost reduction. These two office concepts provide a cost–benefit from a short-term perspective. Fostering interaction and collaboration is another reason to introduce OPO and ABW. However, studies show a decrease in face-to-face interaction in OPO. Furthermore, ABW facilitates interaction and collaboration only when proper space and time management are implemented. Office concepts that meet the needs of employees will avoid the productivity tax of malfunctioning “modern” office concepts.

### Limitations and future research

This study has several limitations that open fruitful areas for further research. First, only English papers are included, as English is the standard international language in peer-reviewed publications. However, including articles in other languages could broaden the scope of an SLR (Jurecic 2020; Krupper 2011; Pauls and Lütke Lanfer 2017). Second, as research on the consequences of office architecture on individual and organizational performance has been neglected in management studies, this SLR covers a wide range of disciplines, including papers from various adjacent fields. Consequently, the journal quality of the included articles is not comparable and may vary. However, it was particularly important to conduct an SLR as a first step to provide a first integrative overview. We encourage scholars to build on this SLR and contribute to this fertile field (with particular emphasis on management studies). Third, this research uses performance as an umbrella term that includes quality and quantity of outcomes because our objective is to identify the differences between the three office concepts rather than to analyze the effects of these office concepts individually. This is since the existing state of research does not allow for such a fine-grained analysis. A distinction between productivity, efficiency, and performance measurements would be useful for scholars and practitioners alike. Fourth, office concepts may also affect other organizational performance values, such as innovation and flexibility. It would be interesting to research how different office concepts affect an organization's innovation performance, as these are the current challenges of most industries. Fifth, this SLR only briefly touches on remote work, an emerging work concept that will gain importance, especially considering COVID-19. Employees will be in the office less often. Probably, team meetings and face-to-face interaction will gain more importance in the office space at the company. This will affect physical office requirements. Additionally, the remote office is within a private setting. Thus, feelings like dehumanization will not play a role. However, other aspects will arise: guaranteeing a professional workplace, being able to distinguish between professional and private time, and the rise of other distraction levels (children, partners, etc.), among others. Some corporations proclaim to become ‘all remote’. “Companies including Pinterest, Dropbox, Twitter, and Yelp have charted out a hard pivot from swanky, perk-filled offices to embrace remote-working futures, while larger firms, like Facebook and Salesforce, have said they plan on more flexible schedules”, says Gregory Barber in Wired Magazine (Barber 2021). These companies will be at the forefront of becoming virtual institutions. It may be possible that companies will operate without any headquarters, even without any office, as all their employees will work remotely. With companies that market products without running their own production sites, such as Nike as a role model, a new type of all-remote corporation has become the vision for some corporate leaders and would cause a significant shift in our work environments. However, already in 2017, IBM ended the era of remote working, although the earlier remote policy had resulted in significant cost reduction: an office space reduction of 78 million square feet that led to a cost saving of over 100 million US dollars (Kessler 2017). But they found that employees work more effectively and experience higher job satisfaction when working shoulder to shoulder, so the IBM workforce was moved back to offices. According to John Sullivan, a professor of management at San Francisco State University who specializes in HR strategy working together in person is one key to innovation (Kessler 2017). Most recently, Elon Musk followed the IBM example by ordering all Tesla employees back to their (physical) offices post-COVID (McGahey 2022). The study of office concepts from the perspective of remote work is a promising avenue for future researchers (Table 2).

**Table 2:** Taxonomy of office concepts

Term	Definition
Single office	One employee works alone in his/her private room
Shared room office	Two or three employees share a single room
Small OPO (open-plan office)	Four to nine employees share a room
Medium-sized OPO	10 to 24 employees share a room
Large OPO	More than 24 employees share a room
ABW (activity-based working)	Employees can choose from various workstations (often including an OPO environment as well as quiet work areas)

This SLR provides a concise overview of the existing body of research and names knowledge gaps to be investigated in future research that will deepen and extend our understanding of office concepts and their impact on performance (see Table 3). Table 3 supplies a brief overview of the state of the art, and Table 4 provides selected opportunities for future research. In this section, we broaden our view and show other areas that will benefit from further research. This SLR reveals that current research did not differentiate between distinct types of knowledge workers. A more precise look will be fruitful, as different knowledge work calls for specific environments. One may imagine that, for instance, one type of knowledge work requires data as input that can be displayed on screens, while another requires physical stimulus material scattered around in the office (e.g., financial analyst versus textile designer). The different work tasks of knowledge workers will be reflected in future research. Research may also examine the rhythms of knowledge work: how often and when periods of concentrated work are prevalent compared to periods of sharing ideas with coworkers. In addition, this SLR found a research gap on personality traits in general. We suggest studying how different office concepts influence the performance of different personality types, thus helping to design office environments that support personal satisfaction, health, and performance. This leads to future research on team characteristics. What is the best match of office concept and team, e.g., what is the ideal office concept for teams that occasionally meet for a quick exchange, and what kind of office suits best a team that works together over a prolonged period of time in a concentrated manner? This train of thought leads to interaction and cooperation. What are the differences between concentration and cooperation and concentration and creativity, and how can these issues be addressed in an appropriate office concept? We see exciting potential in interdisciplinary research teams that can integrate/merge different research fields, e.g. psychology, management, and architecture. Regarding the successful implementation process of new office concepts, scholars have not examined, to date, the interaction of an office concept with the organizational management styles. It is reasonable to assume that office concepts and management styles affect each other. What percent of changes in this quality can be accounted for by the physical design, and what percentage is due to managerial influences? Thus, research may carefully investigate both ways: how brick and mortar affect management and how certain management styles lead to specific architecture (think, for instance, about Google vs. Apple). In addition, longitudinal studies are needed to investigate the habitual effects of office concepts. How do occupants learn to use a specific office concept? Last, but not least, office concepts are perceived as a signal in the “war of talents”: There may be differences between employee attraction (short-term effects during the application phase) and employee retention (long-term effects in the daily life of employees). Here, we again face competing goals of realizing quick short-term success versus ensuring long-term success.

**Table 3:** State of the Art

Office concept (transitions)	Dimension					
	Work	Personality fit	Satisfaction	Health	Enabling paradigm	Control paradigm
Traditional office to open-plan office	**–**	**?**	**–**	**–**	**–**	** + **
Individual office to open-plan office	**?**	**?**	**–**	**–**	**–**	**?**
Open-plan office	** ~ **	** ~ **	**–**	**–**	**–**	** + **
Open-plan office to activity-based working	** ~ **	**?**	** + **	** + **	** ~ **	** + **
Traditional office to activity-based working	** ~ **	**?**	**–**	** + **	**–**	** + **
Diverse offices to activity-based working	** ~ **	**?**	** ~ **	** + **	** ~ **	** + **
Activity-based working	** ~ **	**?**	** ~ **	** ~ **	** ~ **	** + **

** + **positive effect, **–** negative effect, ~ highly context-specific, **? **not enough evidence

**Table 4:** Selected opportunities for future research

Elements	Research gaps	Research question examples
Office concept	Comparison between traditional office settings to ABW	R1: How do employees react with respect to satisfaction, health and performance when moving from a traditional office setting (with single offices and meeting rooms) to ABW (including quiet work zones and collaboration areas)?
	The implementation process (of ABW)	R2: What are the key drivers of successful ABW implementation?
	Management systems of ABW	R3: How do different space and time management systems of ABW impact occupants' work, satisfaction, health, and performance?
Level of distraction	The design of quiet work areas	R4: How should quiet work areas designed? What exactly do occupants look for when looking for a quiet work area?
Personality Fit	Personal preferences and work typologies	R5: How do different personality types cope and adapt to different office concepts in relation to the type of (knowledge) work?
Control Paradigm	Measurement of real estate cost across different areas	R6: How do organizations measure real estate costs across different areas (e.g., San Francisco financial district vs. Minnetonka in Minnesota)?
Enabling Paradigm	Measurement of workers' productivity	R7: How can we measure the workers' productivity in different office concepts?
	Drivers of innovativeness	R8: What office concept enhances innovation?
	Measurement of productivity tax	R9: Develop a model that measures the productivity tax by defining the KPIs for each outcome variable

### Practical implications

The current policy of implementing office concepts neglects the individual needs of employees and causes a sense of feeling dehumanized. Traditionally, office space is perceived as an expenditure that should be minimized. This SLR suggests looking at office space as a room for human development. First, the workspace should match the demands of the job. Second, there is a need for quiet work areas, as well as areas for collaboration, especially when different personality types are considered. Offer a proper mix of areas based on work tasks and personalities for each office location. Furthermore, planning an office concept is an impactful strategic management tool because one decides who will meet and how often in which environment due to physical proximity. Third, noise distraction is an undesired side effect of open workplaces such as OPO and ABW. The noise level should never be higher than 48 dB(A). Install noise reduction elements in open areas or minimize unbounded areas. Fourth, workers prefer to have control over their workspace. Corporate policies should allow employees to personalize their workspace. Fifth, ABW is not an architectural solution but a management task. Implement a time and space management system that meets the needs of employees. Sixth, despite a trend to OPO and ABW, the single office has its merits: Employees have the most control over their office environment, including light, noise, interaction, aesthetics, etc. Despite its name, the single office allows interaction and collaboration when needed (open door/closed door). Organizations may examine which office concept best suits the needs of its employees and not disregard the single office as old-fashioned upfront. Seventh, the office concept reflects corporate culture; organizations are well advised to choose an office concept that nourishes a human-centered culture. Even from a managerial perspective, a good office concept helps to keep the retention rate high and attracts new employees. As a final remark, designing “the optimal office concept” seems to be a constant work in progress. We encourage to build interdisciplinary teams consisting of but are not limited to managers who understand and plan the workflow and have a clear perspective on performance criteria, real estate planners who have an eye on cost, architects, who know how to build attractive spaces, psychologist, who focus on physical and mental well-being and motivation, and, most of all, workers who will actually work in the planned office. Ideally, the latter will be the focal point of all planning and designing. We could also envision a ‘mock-up’-phase, where the office is already occupied, but certain elements are still in flux (see, for instance, the architect Alexander (2002) developed design patterns and a way of building that indicate future paths to office designs). Both the organization and its workers will benefit from a holistic and human-centered office concept.

## Data Availability

All data generated or analyzed during this study are included in this published article [and its supplementary information files].

## Appendix A

Summary of reviewed papers.

**Table:**

Author	Year	Sample *n* =	Aim of study	Key findings
*From traditional to Open-plan office*				
Baldry and Barnes	2012	18	Examine the introduction of OPO	Cost reduction is a main reason for OPO
				Single offices are important for self-esteem, professional identity, appreciation for employees and overall image
Bernstein and Turban	2018	152	Examine the effect of open office architectures on employees’ face-to-face, e-mail and instant messaging interaction patterns	Decrease in F2F communication
				Increase in electronic communication
				Decrease in productivity*
Di Blasio et al.	2019	1078	Study the introduction of OPO	Increase noise and stress
				Decrease in mental health symptoms and well-being
				Decrease in productivity
Kaarlela-Tuomaala	2009	31	Examine how the perceived acoustic environment differ from single office to OPO	After relocating to OPO:
				Increase noise distraction and concentration problems
				Reduced privacy
				Reduced interaction
Kim and De Dear	2013	42,764	Examine if OPOs facilitate communication, interaction and satisfaction	Enclosed private offices outperform OPOs in almost all aspects (IEQ*)
Sailer and Thomas	2021	3 organizations	Examine socio-spatial perspectives on OPO versus traditional offices	All three organizations have different demands on their office concept and on interaction
				The most unplanned interaction was found in traditional office
Sander et al.	2021	43	Compare effects of noise in simulated OPO with quieter private office environment	In OPO: decrease in psychological well-being (increase in stress)
*From individual to Open-plan office*				
Bergström et al.	2015	79	Investigate perceived health, work environment and self-estimated productivity one month before and at three, six and twelve months after relocation from individual offices to an open-plan office environment	Decrease in satisfaction
				Decrease in health
				Decrease in productivity*
*Open-plan office*				
Ahmadpoor et al.	2017	238	Assess the impact of employees' perceptions of workplace design and conditions on their performance and behavior	Decrease in satisfaction
				Decrease in productivity*
				Increase in team interaction
Chacon et al.	2020	3028	Investigate the performance of OPO layouts and occupants’ concerns	Physical environment influences satisfaction
				Need for areas for quiet working
Chadburn et al.	2017	213	Analyze the drivers that allow for enhanced personal productivity of knowledge-based workers	Office design influences knowledge workers productivity
				Need for interaction areas as well as quiet zones
Costa and Villarouco	2012	3 departments	Systematically analyze the OPO working space	Office layout must follow the characteristics of tasks and their interrelationships (since adverse physical and organizational conditions may have a negative impact on work productivity)
				To increase productivity the worker’s health and work efficiency must be ensured
Göçer et al.	2012	2133	Difference between high vs low performance OPOs	Aesthetics and quality, noise distraction and privacy are the strongest predictors for low performance OPOs
Muville et al.	2016	1 building	Examine the impact of the ambient environment on perceived comfort, health, well-being and by extension, productivity in the workplace	Significant differences in ambient environment can exist in relation to comfort, health, well-being and by extension productivity within individual buildings
				No measurable differences in directly related ambient
Oseland and Hodsman	2020	2145	Identify personal factors, including personality traits, which underpin noise distraction of office workers in order to mitigate noise and improve well-being and performance	Noise distraction depends on job roles and personality
				Noise affects stress and well-being
Roelofsen	2008	n.a	Examines how productivity* can be negatively affected in OPO environment	The ideal background noise level of OPO is between 45 dB(A) and 48 dB(A)
				Decrease in productivity* can be related to speech intelligibility
				Need for smaller closed office spaces for telephone calls, accurate or creative work
				Personnel costs higher than real estate costs
Roskams and Haynes	2020	78	Test employee’s satisfaction within the work environment	Higher environmental comfort was associated with improved well-being and productivity*
				Distraction leads to less productivity
Samani and Alavi	2020	117	Influence of open‐plan workplaces on employees' work‐related behavior and their outcome	Increase in a more democratic working culture
				Increase in interaction
Samani et al.	2017	238	How individual perception affects creative outcome in OPOs	Personal control over work environment must be ensured to raise social interaction and creative outcome
Seddigh et al.	2015	527	Effect of office design and performance on cognitive tasks	Increase productivity* in calm work environments
Veitch et al.		779	Develop a model linking environmental and job satisfaction (OPO)	OPO occupants who were more satisfied with their environments were also more satisfied with their jobs, suggesting a role for the physical environment in organizational well-being and effectiveness
Węziak-Białowolska et al.	2018	456	Aim to understand the relationship between perception of the OPO and the impact on employees as well as on their behavior	Decrease in privacy and a high office density affect satisfaction
*From open-plan office to activity-based working*				
Blok et al.	2009	684	Effect of work environment on communication, concentration and productivity	Less costs than OPO
				Increase in satisfaction, team interaction, productivity and concentration
Candido et al.	2021	2090	Study predictors of occupants’ satisfaction and productivity of five different industries	Interior design and physical configuration play a crucial role on occupants’ satisfaction and productivity
Candido et al.	2019	896 + 20	Effect before and after relocation	Increase in satisfaction, health, IEQ and productivity*
Divett	2020	1275 + 138	Evaluates satisfaction and productivity	Increase in satisfaction, interaction and productivity*
				Key drivers of productivity are team interaction and decision-making
Foley et al.	2016	88	Sedentary behavior and musculoskeletal discomfort in ABW	Increase in health (standing time, less lower back pain)
Rolfö et al.	2018	34 + 66	Explore how physical conditions, office use, communication, privacy, territoriality, satisfaction and perceived performance change following a company’s relocation from an OPO to an ABW	Increase satisfaction compared to OPO
				No change in productivity*
				Lack of communication and rules lead to high people-to-workstation ratio
*From traditional office to activity-based working*				
Arundell et al.	2018	146	Examine the influence of redesign office concept	Increase in physical activity, interaction with colleagues
				Positive effect on eating behavior
				Small decrease in productivity
Gorgievski et al.	2010	266	Examine a post-occupancy evaluation	Lack of privacy
				No storage for personal stuff
				Anonymous workplace
				Increase in working at home
*From diverse offices to activity-based working*				
Brunia et al.	2016	930	Analyze accommodation of new office concepts	Employee satisfaction is influenced by many physical characteristics of the work environment and by the implementation process
Keeling et al.	2015	179	Analyze the effect of agile workspace	Agile working renegotiates the trade-off between interaction and privacy
				Increase in interaction and privacy compared to OPO
*Activity-based working*				
Appel-Meulenbroek et al.	2011	182	Relationship between office design, its intentions and the actual use after implementation	When ABW is not used as intended: decrease in satisfaction, health and productivity*
Haapakangas et al.	2018	239	Determine perception and use of ABW in relation to self-rated productivity and well-being at work, and to identify important predictors of these outcomes	Work environment influences satisfaction and productivity*
				Privacy and communication are important for productivity*
				Need for minimization of work time spent looking for available workspaces
Fincke et al.	2020	16	Analyze job demands and resources of ABW	Work autonomy, the flexibility to decide where and when to work, and an improved communication and collaboration between different departments have a perceived positive effect on well-being, performance and motivation
Nijp et al.	2016	2912	Examine the effects of NWW on organization and work and on employee’s outcome	Does not necessary lead to changes in psychosocial work characteristics
				Decrease in health (intervention group)
Parker	2016	n.a	Examine the phenomenon of activity-based working	Key output for ABW: Cost efficiencies and productivity. While ABW adopters and advocates present ABW as a desirable staff satisfaction and operations facilitator, the cost efficiency, however, is pre-dominant
Wadu and Chiang	2019	37 + 3	Impact on employee productivity of adopting ABW	Distraction leads to less productivity*
				Physical and *behavioral* (more) working environmental factors influence employee productivity
*Various office concepts*				
Appel-Meulenbroek et al.	2020	150	Study occupants’ choice of coping strategies regarding different sources of noise	Approach coping strategy is less often chosen than avoidance strategy, although higher impact is expected
				Choosing a specific coping strategy depends on the source of noise
Banburry and Berry	2005	88	Examine noise distraction and concentration problems in offices	Out of the sample, 99% participants report about concentration problems
Danielsson et al.	2008	469	Examine health and satisfaction in different office concepts	OPO: lowest health
				Cell and flex offices: best health
				Goes along with job satisfaction
De Been and Beijer	2014	11,799	Determine whether the type of office concept has an impact on satisfaction with the office environment and productivity support	Occupants of combi office and ABW report less productivity support, less concentration and privacy than people of single office or shared office
Haynes et al.	2019	405	Explore the relationship between office occupier work activity and workplace provision	A greater level of location-flexibility lead to more productivity*
				ABW is good for collaborative tasks
				Location-fixed workers: decrease in IEQ, especially noise distraction
Kwon and Remøy	2020	579	Identify the weight of contribution of each design parameter on increasing psychological satisfaction	Cellular office north-west oriented workstation and 4 m away from a window optimal for satisfaction
Pejtersen et al.	2011	2403	Examine sick absence in OPO, shared and cellular offices	Occupants of shared offices and OPO have significantly more days of sickness absence than occupants of single offices
Taskin et al.	2019	534 + 12	Analyze the dark side of office concepts	Influence on satisfaction and productivity varies on different office concepts
				The more the office design is anonymous, the more the worker feels de‐humanized by his/her organization

Productivity* = productivity or efficiency or performance
